# Impute the missing data using retrieved dropouts

**DOI:** 10.1186/s12874-022-01509-9

**Published:** 2022-03-27

**Authors:** Shuai Wang, Haoyan Hu

**Affiliations:** 1 Global Product Development, Pfizer Inc, Groton, CT 06340 USA; 2 Department of Statistics, Iowa State University, Ames, IA 50011 USA

**Keywords:** Missing not at random, Multiple imputation, Treatment policy, ICH E9 (R1), Retrieved dropouts

## Abstract

**Background:**

In the past few decades various methods have been proposed to handle missing data of clinical studies, so as to assess the robustness of primary results. Some of the methods are based on the assumption of missing at random (MAR) which assumes subjects who discontinue the treatment will maintain the treatment effect after discontinuation. The agency, however, has expressed concern over methods based on this overly optimistic assumption, because it hardly holds for subjects discontinuing the investigational drug. Although in recent years a good number of sensitivity analyses based on missing not at random (MNAR) assumptions have been proposed, some use very conservative assumption on which it might be hard for sponsors and regulators to reach common ground.

**Methods:**

Here we propose a multiple imputation method targeting at “treatment policy” estimand based on the MNAR assumption. This method can be used as the primary analysis, in addition to serving as a sensitivity analysis. It imputes missing data using information from retrieved dropouts defined as subjects who remain in the study despite occurrence of intercurrent events. Then imputed data long with completers and retrieved dropouts are analyzed altogether and finally multiple results are summarized into a single estimate. According to definition in ICH E9 (R1), this proposed approach fully aligns with the treatment policy estimand but its assumption is much more realistic and reasonable.

**Results:**

Our approach has well controlled type I error rate with no loss of power. As expected, the effect size estimates take into account any dilution effect contributed by retrieved dropouts, conforming to the MNAR assumption.

**Conclusions:**

Although multiple imputation approaches are always used as sensitivity analyses, this multiple imputation approach can be used as primary analysis for trials with sufficient retrieved dropouts or trials designed to collect retrieved dropouts.

**Supplementary Information:**

The online version contains supplementary material available at 10.1186/s12874-022-01509-9.

## Background

In a randomized trial with a continuous primary endpoint, usually multiple visits are scheduled after the screening and randomization visits. For instance, in a 26-week type 2 diabetes (T2D) randomized trial, Week 6, 12, 18 and 26 are scheduled as post-baseline visits. Change from baseline in A1c (%) at Week 26 is chosen to be the primary endpoint, because A1c has been chosen as the primary biomarker by regulatory agencies in evaluating treatment effect of antidiabetic medications since 1990 [1, 2], according to published guidelines [3]. Although every effort has been made to collect data and keep subjects remain in the trial, missing data still occurs inevitably [4] due to different reasons: lost to follow-up, withdrawal by consent, subjects move, site closure, collection error, missed visits and so on. When it comes to analyzing a continuous endpoint in a longitudinal setting, the most commonly used primary statistical method is mixed models repeated measurements (MMRM) or its variations like constrained longitudinal data analysis (cLDA) [5]. This type of methods accounts for missing data in an implicit fashion that imputation of missing data is not needed due to its underlying assumption of MAR. Since such assumption usually doesn’t hold in a real clinical trial, sponsors are also required to provide additional sensitivity analyses with missing data imputed based on the MNAR assumption [6, 7], as further evidences to support the robustness of the primary conclusion. In terms of implementation, some sensitivity analyses are more complex requiring more computer resources than others (e.g. jump to reference based on multiple imputation [8, 9] vs. Last observation carried forward based on single imputation [10]). In the past few years, multiple-imputation (MI) based methods have been gaining more popularity and increasingly requested by regulatory agencies, because it can handle more complex or user-defined distribution/assumption in the imputation. It’s well known that MI analyses based on MAR assumptions treat missing data as ignorable and therefore the impact of such implementations is questionable. Implementations based on MNAR assumptions are more widely used and acceptable from regulatory perspective [6]. Little first proposed pattern mixture model [11–13], a broad class of methods imputing missing data mainly based on MNAR. More specifically, each implementation is mainly driven by its underlying assumption. For instance, if the missing data in the active group is assumed to have the same distribution as the control group after the subject’s discontinuation, jump to reference (J2R) or its variations [8, 9, 14] such as copy reference, copy increments in reference will apply. Whereas methods assuming that the treatment effect is expected to wash out after the subject’s discontinuation, and return to the baseline level, correspond to return to baseline (RTB) [15] or baseline observation carried forward (BOCF) [1–6, 8–10, 14–18]. Anther class of methods that have been proved useful is tipping point MI analyses [13, 19, 20] which use stress testing strategies to identify tipping point by gradually increasing and adding MNAR penalties (i.e. more and more unfavorable to the investigational product) to data imputed under MAR, until statistical significance vanishes. It usually needs to be interpreted under clinical context of the endpoint of interest, and a very large tipping point usually is a good indication of robust primary results. There have been debates on the rise between sponsor and regulatory agencies as which MNAR assumption should be used for a specific study: some of the very “conservative” approaches such as J2R, RTB will help regulators understand if the drug will still work under very unfavorable assumptions, but on the other hand it might not be convincing to have sponsors agree that these estimates should be used for drug labelling given that certain extreme assumptions might not hold for some missing data.

The approach we propose should serve as a good “trade-off” between “conservative” MNAR and “optimistic” MAR assumptions. It assumes subjects who discontinue the trial tend to have similar values on the endpoint, compared to those in the same treatment group who are already off treatment but remain in the study (“retrieved dropouts”) after adjustment of certain baseline covariates and last on-treatment visit. The concept of “Retrieved dropouts (RDs)” was first described as subjects with data collected after cessation of study treatment in the published guideline “Missing data in confirmatory clinical trials” [6] in 2010, but the guidance didn’t provide technical details of implementation. Several sponsors have estimated the difference of treatment effect solely based on RDs in their post-hoc analyses [21]. Chen and colleagues [22] proposed a Bayesian method to estimate the difference of treatment effect for each subset of the population including off-protocol subjects. Because their method couldn’t generate an estimate of the overall treatment effect difference as well as lack of type-I error validation, it has limited value in real application of clinical trials. Pampaka [23] imputed the missing data using RDs along with completers which on one hand led to better imputation precision, but on the other hand might be overly optimistic to assume the treatment effect of missing data follows the distribution of pooled data of RDs and completers.

Our approach doesn’t have such obstacles. The basis of the multiple imputation in our approach is RDs defined as subjects off treatment but still in the study and have the primary visit measurements available. As for predictors of this regression-based multiple imputation, at least baseline and last on-treatment visit should be included for which more justifications are provided in the discussion section. A minimum of 100 imputations are recommended as more imputations can effectively prevent power falloff for small effect size [24]. Then each full dataset will be analyzed using analysis of covariance (ANCOVA) with baseline value, treatment as well as other pre-specified covariates as covariates. Results from these multiply imputed datasets will then be combined into a single estimate and statistical hypothesis testing can be conducted. From the perspective of estimands [7, 25, 26], this method well aligns with the treatment policy (TP) estimand [26] as described in ICH E9(R1) [7], which includes data collected post occurrence of intercurrent events in the analysis, as opposed to the hypothetical estimand [7, 26] excluding data collected post occurrence of intercurrent events. Treatment policy estimand analyses have been requested by more than one regulatory agency for labelling consideration in recent years [15, 27, 28].

In this manuscript, we first describe the statistical method. Then we will explore and answer the following questions sequentially: 1) which scenarios does this method best apply to; 2) what is the type-I error rate of this method and how is it compared to the commonly used primary and sensitivity analysis methods? 3) what is the power rate of this method, compared to other methods? 4) how to apply this approach to a dataset containing few RDs?

Finally, we will illustrate this method by applying it to a real unblinded dataset from a Phase III lipid-lowering program as a post-hoc analysis.

## Methods

### Statistical methods

Assume a total of N subjects are randomized to two treatment groups (investigational product and placebo) in 1:1 ratio. Let ***Y***_***i***_ denote the longitudinal vector of a continuous primary endpoint for the i-th subject (i = 1, …, *N*), i.e. ***Y***_***i***_ ***=*** (*Y*_*i*0_, *Y*_*i*1_, …, *Y*_*iK*_) if a total of (K + 1) visits are planned including the baseline visit *Y*_*i*0_. *Y*_*iK*_ denote the primary visit. If some visits are missed or results are not available due to reasons such as laboratory sample analysis errors [29] they will be set to missing.

On- and off- treatment visits need to be pre-defined and their definition relies on the endpoint, the half-life of a drug and study design. The population of RDs form the basis of the imputation and is defined as the collection of subjects whose primary visits have occurred off-treatment. Although primary analysis using on-treatment visits based on “hypothetical” estimand [7] has been widely used by sponsors in the past, nowadays regulatory agencies have been increasingly requesting analyses based on the TP estimand [7] to align with the intent to treat (ITT) principle. This method is a good representation of the TP estimand by including RD’s off-treatment primary visits in the analysis because off-treatment visits are considered data collected post occurrence of treatment discontinuation, a type of intercurrent events.

**Fig. 1:**
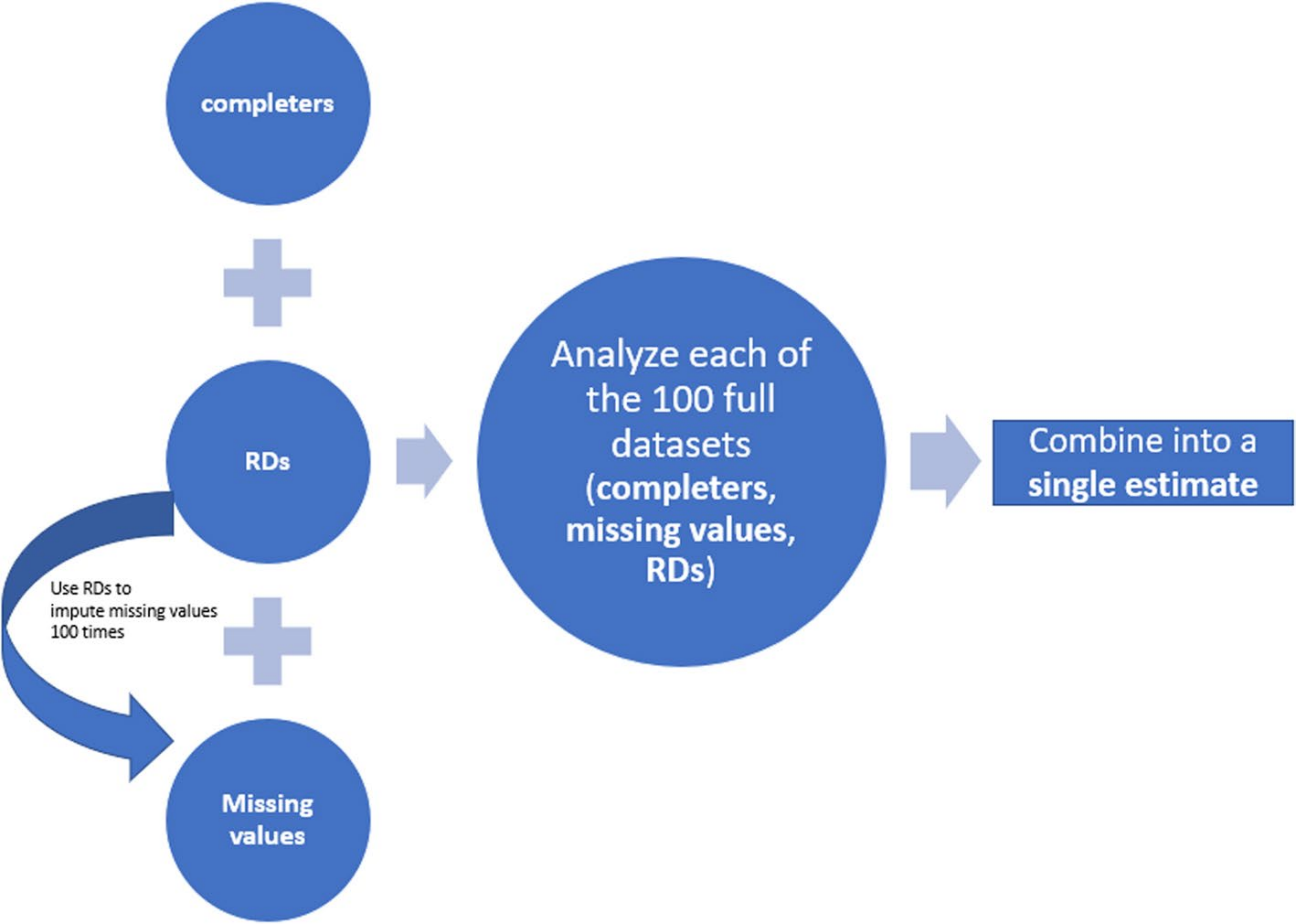
Flow of steps in MI-RD implementation

To better illustrate this method, we decompose a dataset into 3 subsets (Fig. 1): subjects with missing values of the primary visit, denoted by $\boldsymbol{M}=\left\{{\boldsymbol{Y}}_{m_1},\dots, {\boldsymbol{Y}}_{m_{n_{miss}}}\right\}$, RDs (i.e. off-treatment “completers”) denoted by $\boldsymbol{R}=\left\{{\boldsymbol{Y}}_{r_1},\dots, {\boldsymbol{Y}}_{r_{n_{rd}}}\right\}$ and the rest (i.e. on-treatment “completers”) denoted by $\boldsymbol{C}=\left\{{\boldsymbol{Y}}_{c_1},\dots, {\boldsymbol{Y}}_{c_{n_{com}}}\right\}$ where *n*_*miss*_ + *n*_*rd*_ + *n*_*com*_ = *N*. The proposed method consists of three sequential phases: imputation of missing data, estimation and statistical hypothesis testing after combining estimates into a single estimate.

#### Imputation of missing data

The imputation of missing values is based on RDs (i.e. ***R***), but the analysis is based on the full dataset (i.e. ***M ∪ R ∪ C***) regardless of occurrence of intercurrent events. Ideally, the multiple imputation is implemented in groups defined by treatment group and the last on-treatment visit (i.e. among subjects receiving the same treatment and discontinuing the treatment at the same visit). This step requires further decomposition of ***M*** and ***R***. Let ***M = M***_***p***_ ***∪ M***_***d***_ and ***R = R***_***p***_ ***∪ R***_***d***_ where ***p*** and ***d*** represent placebo and investigational drug respectively**.** Then for each of ***M***_***p***_***,M***_***d***_***,R***_***p***_***,R***_***d***_**,** it will be further refined to ***M***_***p***_ ***= M***_*p*1_ ***∪ M***_*p*2_ ***∪*** … ***∪ M***_*pj*_ ***∪*** … ***∪ M***_*pk*_ assuming there are *k* post-baseline visits prior to the primary visit with *j* = 1, …*k* denoting last on-treatment visit. At the imputation step, each subset of ***M*** needs to be paired with the same subset of ***R*** such that they match on the treatment group and last on-treatment visit (i.e. **Ω**_*pj*_ ***= M***_*pj*_ ***∪ R***_*pj*_**, Ω**_*dj*_ ***= M***_*dj*_ ***∪ R***_*dj*_)**.** For instance, there are two treatment groups and subjects’ last on-treatment visit is week 12 or week 18 in a clinical study. Ideally the multiple imputation should be implemented in each of the 4 groups (**Ω**_*pj*_**, Ω**_*dj*_ (*j* = 1, 2)) respectively. The missing data of each **Ω**_***tj***_ (i.e. **M**_***tj***_) are imputed using model constructed from ***R***_*tj*_ (*t* = *p or d*, *j* = 1, …*k*), adjusting for baseline and last on-treatment visit *j* as covariates, written as

1 $$\boldsymbol{Y}={\beta}_{0, tj}^{IMP}+{\beta}_{1, tj}^{IMP}{\boldsymbol{Y}}_b+{\beta}_{2, tj}^{IMP}{\boldsymbol{Y}}_j+\boldsymbol{\varepsilon}$$

where ***Y*** is the endpoint at primary visit, e.g. change from baseline in A1c at Week 26, ***Y***_*b*_ is the baseline, ***Y***_*j*_ denotes visit *j* which is the last on-treatment visit for subjects in **Ω**_***tj***_ and ***ε*** is the random error term.

For better illustration of the imputation phase, the steps are summarized as below:

1. A linear regression model is fit using subjects in ***R***_*tj*_ with the estimated coefficients and mean square error denoted as $\hat{{\boldsymbol{\beta}}^{IMP}}$ and ${\hat{\sigma}}_{tj}^2$.
2. Then for each imputation (*m* = 1, …, *M*) assuming a total of *M* imputations, the regression parameters ${\boldsymbol{\beta}}^{IMP(m)}=\left({\beta}_{0, tj}^{IMP\ (m)},{\beta}_{1, tj}^{IMP\ (m)},{\beta}_{2, tj}^{IMP\ (m)}\right)$ are randomly generated from the posterior predictive distribution of the regression coefficients, i.e. ${\boldsymbol{\beta}}^{IMP(m)}\sim MVN\left(\hat{{\boldsymbol{\beta}}^{IMP}},{\boldsymbol{V}}_{\boldsymbol{tj}}\right)$ where ${\boldsymbol{V}}_{\boldsymbol{tj}}={\left({\boldsymbol{D}}_{tj}^{\prime }{\boldsymbol{D}}_{tj}\right)}^{-\mathbf{1}}{\sigma}_{tj(m)}^2$, ***D***_***tj***_ is the design matrix of the above regression model and ${\sigma}_{tj(m)}^2={\hat{\sigma}}_{tj}^2\left(\#{\boldsymbol{R}}_{tj}-3\right)/{c}_{tj}^{(m)}$ with #***R***_*tj*_ being the sample size of ***R***_*tj*_ and ${c}_{tj}^{(m)}$ being randomly generated from a ${\chi}_{\#{\boldsymbol{R}}_{tj}-\mathbf{3}}^2$ with a degree of freedom of (#***R***_*tj*_ ***−*** **3**).
3. For each subject in **M**_***tj***_**,** the imputed value of *Y* will be calculated using formula (1), ${\sigma}_{tj(m)}^2$**,** ***β***^*IMP*(*m*)^ and *ε* randomly sampled from $N\left(0,{\sigma}_{tj(m)}^2\ \right)$.

#### Estimation

Each set of imputed subjects ${\boldsymbol{M}}^{(m)}={\boldsymbol{M}}_p^{(m)}\cup {\boldsymbol{M}}_d^{(m)}$ (*m* = 1, …, *M*) will be analyzed together with completers ***C*** and RDs ***R*** using ANCOVA adjusting for baseline, treatment group as well as other pre-specified covariates if any, written as

2 $$E\left(\boldsymbol{Y}\right)=\mu +{\beta}_1\boldsymbol{trt}+{\beta}_2{\boldsymbol{Y}}_b$$

Because we are primarily interested in estimation of treatment effect *β*_1_, all *M* sets of ${\left.\hat{\Big({\beta}_1^{(m)}},\mathit{\operatorname{var}}\left(\hat{\beta_1^{(m)}}\right)\Big)\right|}_{m=1,\dots, M}$ will be generated.

#### Statistical hypothesis testing

${\left.\hat{\Big({\beta}_1^{(m)}},\mathit{\operatorname{var}}\left(\hat{\beta_1^{(m)}}\right)\Big)\right|}_{m=1,\dots, M}$ will be combined into a single estimate following Rubin’s rule [30]:

3 $$\hat{\beta_1}=\frac{\sum_{m=1}^M\hat{\beta_1^{(m)}}}{M}$$

The variance of the combined estimate is obtained as $V={V}_w+\left(1+\frac{1}{M}\right){V}_b$ where ${V}_w=\frac{\sum \mathit{\operatorname{var}}\Big(\hat{\beta_1^{(m)}\Big)}}{M}$ and ${V}_b=\frac{\sum {\left(\hat{\beta_1^{(m)}}-\hat{\beta_1}\right)}^2}{M-1}$ are referred to as within- and between-imputation variance respectively. Rubin has demonstrated the test statistic $\frac{\hat{\beta_1}}{\sqrt{V}}$ follows a *t* distribution with a degree of freedom of $\frac{\left(1+\frac{1}{M}\right){V}_b}{V_w}$ under the null hypothesis that *H*_0_ : *β*_1_ = 0. As for the choice of *M*, *M* =100 is recommended because 1) this approach is not computationally intensive using any common statistical software such as SAS or R; 2) more imputations can prevent power falloff [24]; 3) regulatory agencies are generally supportive of 100 multiple imputations for analyses based on multiple imputations.

When the regression-based MI model is not estimable due to non-sufficient RDs in at least one group, say, **Ω**_*p* ∗ *j* ∗ ′_ the *imputation phase* will be simplified and implemented by treatment group only, i.e. within **Ω**_*p*_ ***= M***_*p*_ ***∪ R***_*p*_ and **Ω**_*s*_ respectively. Given **Ω**_*t*_ (*t* = *p or d*), a regression model based on ***R***_*t*_ will be constructed as follows:

4 $$\boldsymbol{Y}={\beta}_{0,t}^{IMP}+{\beta}_{1,t}^{IMP}{\boldsymbol{Y}}_b+{\beta}_{2,t}^{IMP}{\boldsymbol{Y}}_L+\boldsymbol{\varepsilon}$$

where ***Y***_*L*_ denotes the last on-treatment visit, concatenated from ***Y***_*j*_ of ***R***_*tj*_ (*j* = 1, …*k*). Then similar to the imputation steps above, ${\left.{\boldsymbol{\beta}}^{IMP(m)}=\left({\beta}_{0,t}^{IMP\ (m)},{\beta}_{1,t}^{IMP\ (m)},{\beta}_{2,t}^{IMP\ (m)}\right)\right|}_{m=1,\dots, M}$ will be sampled from posterior predictive distribution of the regression coefficients so that subjects in ***M***_*t*_ will be imputed. The same *estimation* and *statistical hypothesis testing* procedures that have been described will then follow.

Obviously not every study is designed to collect retrieved dropouts. To make our proposed approach more generally applicable, we will implement this simplified *imputation phase* by treatment group in all subsequent sections. In the MI implementation, the coefficients are randomly drawn from the posterior distribution of the regression coefficients and a large value of mean squared error (MSE) can lead to imputed values out of range, e.g. a negative imputed value is certainly inappropriate for a positive continuous endpoint, therefore it’s no longer a trivial n > p problem in regression models. We will explore and answer the question that at least how many RDs are considered sufficient in part 1 of simulation studies. Since post-processing such as truncation or using truncated normal regression might cause biased estimates of marginal mean when data are highly skewed [31], we won’t impose explicit post-processing steps in the simulations (except for section “No enough RDs”).

### Simulation studies

We simulated a 26-week two-armed clinical trial with 1:1 allocation ratio to placebo or antidiabetic medication. In addition to baseline, 4 post-baseline visits were simulated: Week 6, 12, 18 and 26 among which Week 26 was the primary visit. The endpoint of interest was defined as the change in A1c (%) from baseline at Week 26 [32–35]. Using the formula below, longitudinal A1c (%) values was simulated from a cLDA model [5], assuming the mean of A1c was the same between the two groups at baseline. Visits and treatment group were treated as categorical, with ***β***_0_ =(8.25, 8.25, 8.25, 8.25, 8.25)′ and 8.25 denoting the mean A1c (%) at baseline.

5 $${\boldsymbol{Y}}_i={\boldsymbol{\beta}}_0+{\boldsymbol{\beta}}_t+{\boldsymbol{\beta}}_I{trt}_i+{\boldsymbol{\varepsilon}}_i$$

***β***_*t*_ = (0, −0.01, −0.05, −0.1, −0.2)′ denoted the main effect of visit, i.e. change from baseline at all time points for the reference treatment group and ***β***_*I*_, the interaction term of treatment and visit, representing the difference of treatment effect between the test and reference treatment groups over time would be specified in the following sections. The first element of both ***β***_*t*_ and ***β***_*I*_ were set to 0 due to the correspondence with baseline. *trt*_*i*_ was the treatment assigned to the i-th subject (1 = active; 0 = placebo). ***ε***_***i***_ was the error term to account for the correlation among visits of the i-th subject (i = 1, …, N), which had a multivariate normal distribution of *N*(**0**, **Σ**) based on knowledge from a completed T2D study of a SGLT-2 inhibitor [35] (the diagonal elements were all set to 1, and the rest were all set to 0.6). ***Y***_*i*_ was the vector consisting of absolute A1c values from baseline to Week 26 of the i-th subject (i = 1, …, *N*).

Since not every clinical study is designed to collect RDs, the MI-RD approach in this manuscript is implemented in the less granular way (4) with baseline and last on-treatment visit used as predictors in the regression-based MI.

The simulation studies consist of the following 4 parts: in part 1, the minimum number of RDs is identified for each scenario and then used as input for type-I error simulations (part 2) and power simulations (part 3). In part 4, various strategies on how to handle non-sufficient RDs are explored and compared.

#### Part 1. Best applicable scenarios

The following effect size, ***β***_***I***_, representing difference of treatment effect at all time points were considered. 10, 20, 30, 40, 50 missing per Arm were explored and simulated. Retrieved dropouts’ last on-treatment visit was randomly selected from Week 6, 12 or 18. In this section, we want to answer the following question: given the absolute amount of missingness per arm, at least how many RDs are needed, so that all imputed values are within appropriate range (i.e. 3% [36, 37] < A1c < 15%)?

$$a. {\boldsymbol{\beta}}_{\boldsymbol{I}}=\left(0,-0.05,-0.1,-0.2,-0.25\right)^{\prime }$$

$$b. {\boldsymbol{\beta}}_{\boldsymbol{I}}=\left(0,-0.1,-0.2,-0.4,-0.5\right)^{\prime }$$

$$c. {\boldsymbol{\beta}}_{\boldsymbol{I}}=\left(0,0,0,0,0\right)^{\prime }$$

For every scenario, a total of 5000 simulated datasets were generated. Since scenario a. and b. were simulated based on the assumption that the active group was superior to placebo, RDs of the active group were assumed to have an additional average increase/worsening of 0.25 in A1c at Week 26 compared to completers of the same treatment group due to off-treatment period, i.e. ***β***_***MNAR***_ ***=*** (0, 0, 0, 0,  0.25)^′^

$${\boldsymbol{Y}}_{\boldsymbol{i}}={\boldsymbol{\beta}}_{\mathbf{0}}+{\boldsymbol{\beta}}_{\boldsymbol{t}}+{\boldsymbol{\beta}}_{\boldsymbol{I}}{trt}_i+{\boldsymbol{\beta}}_{\boldsymbol{MNAR}}{trt}_iI\left\{i\ is\ RD\right\}+{\boldsymbol{\varepsilon}}_{\boldsymbol{i}}$$

Where the indicator function *I* was 1 if the i-th subject was an RD, otherwise was 0. Such explicit adjustments were not made to other post-discontinuation visits because those visits were not used in either imputation or analysis. Considering MAR assumption generally holds in placebo group, such explicit adjustments were not applied to placebo RDs.

**Fig. 2:**
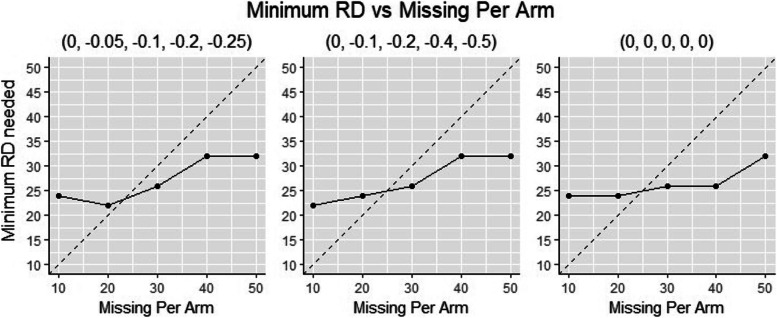
How Minimum Number of Rds Correspond with Absolute Number of Missing per Arm: *the one on the left, middle and right correspond to effect size a, b, c*

A line search strategy on number of RDs was implemented, to identify the minimum number of RDs needed per arm. The criterion was to locate the minimum number of RDs with which the imputed values of all 5000 simulations are within defined range. The results were summarized in results section (Fig. 2, Table 1).

**Table 1:** Minimum Number of RDs Derived from Simulations of Best Applicable Scenarios

Number of Subjects missing Week 26 per Arm *n*_*M*_	Minimum Number of RDs per Arm *n*_*R*_	Number of subjects missing Week 26 Per Arm (Methods except MI-RD) *n*_*M*_ + *n*_*R*_
10	24	34
20	24	44
30	26	56
40	32	72
50	32	82

#### Part 2. Type-I error

The maximum of the minimum RDs across scenario a-c was used as input for Part 3–4, given the number of subjects missing Week 26 per arm (column 2 of Table 1). We evaluated the type-I error rate of a number of different scenarios (sample size, absolute amount of missing data and effect size) as summarized in Table 2. The following two effect sizes were explored assuming there was no difference of treatment effect at Week 26 (i.e. data were simulated under the null hypothesis):

1. no difference of treatment effect between the two treatment groups at all time points:
  $${\boldsymbol{\beta}}_{\boldsymbol{I}}={\left(0,0,0,0,0\right)}^{\prime }$$
2. no difference of treatment effect between the two treatment groups only at Week 26:
  $${\boldsymbol{\beta}}_{\boldsymbol{I}}=\left(0,-0.2,-0.4,-0.8,0\right)^{\prime }$$

**Table 2:** Sample Size Scenarios of Type-I Error and Power Simulations

Number of Subjects missing Week 26 per Arm	Minimum Number of RDs per Arm	Number of subjects missing Week 26 Per Arm (Methods except MI-RD)	Missing rate (methods except MI-RD) 150 subjects per arm	Missing rate (methods except MI-RD) 200 subjects per arm	Missing rate (methods except MI-RD) 300 subjects per arm	Missing rate (methods except MI-RD) 400 subjects per arm
10	24	34	0.23	0.17	0.11	0.09
20	24	44	0.29	0.22	0.15	0.11
30	26	56	0.37	0.28	0.19	0.14
40	32	72	0.48	0.36	0.24	0.18
50	32	82	0.55	0.41	0.27	0.21

**Fig. 3:**
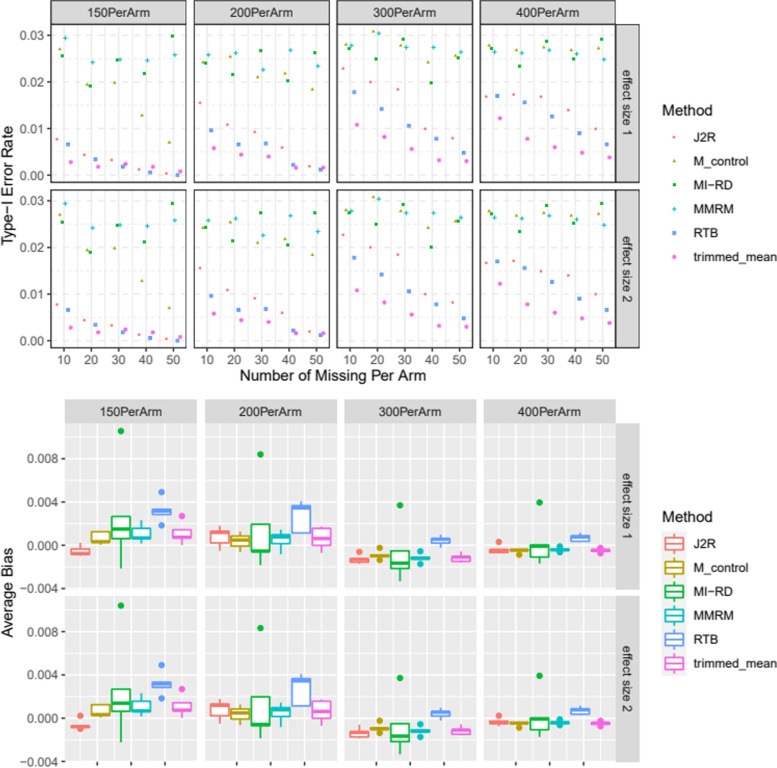
Type-I error rate and average bias of all 6 methods with respect to different sample size, missingness and effect size: M_control denotes Mehrotra’s control-based method; effect size 1 and 2 denote the effect size 1) and 2)

We also considered 4 different sample size N ranging from 150 to 400 per Arm (Table 2) for good representation of different missing rate. In each scenario (N, amount of missing data, ***β***_***I***_), 5000 datasets were simulated. The type-I error rate was defined as the proportion of simulations with one-sided *p*-value significant at *α* = 0.025. Since at Week 26 subjects in the two treatment groups were simulated from the same normal distribution and MAR was assumed for subjects discontinuing the two treatment groups at Week 26, no additional worsening was applied to the off-treatment Week 26 values of RDs in either group. i.e. ***Y***_***i***_ = ***β***_**0**_ + ***β***_***t***_ + ***β***_***I***_*trt*_*i*_ + ***ε***_***i***_**.** This method was compared to a couple of methods very commonly used as primary or sensitivity analyses in clinical trials: MMRM (using compound symmetry as the covariance structure), RTB [15], J2R [14], the adaptive trimming of trimmed means [38] and Mehrotra’s control-based method [39], with the off-treatment Week 26 values of RDs treated as missing in all methods except MI-RD (Tables 3 summarizes how missing values were handled). Baseline eGFR levels were simulated from the eGFR distribution of Vertis CV [40] and was adjusted for along with baseline A1c level in all models. One hundred imputations were utilized in all MI-based methods. The type-I error simulation results were summarized in Results section and Fig. 3.

**Table 3:** Comparison of methods

Method	Missing data	Imputation of missing data
MI-RD	Off-treatment visits are used	Yes using MI
MMRM	Off-treatment visits are set to missing	No
RTB	Off-treatment Week 26 values are set to missing.	Yes using MI
J2R	Off-treatment visits are set to missing	Yes using MI
Trimmed means	Off-treatment Week 26 values are set to missing.	No
Mehrotra’s control-based	Off-treatment Week 26 values are set to missing.	No

##### RTB

Missing Week 26 value is imputed using normal distribution with baseline value as the mean and the mean square error (MSE) from ANCOVA model based on completers as variance. Its underlying assumption is that subjects who discontinue from treatment will experience a washout of treatment effect and therefore their value will eventually return to baseline level.

$${Y}_{j5}\sim \boldsymbol{N}\left({Y}_{j1},{\sigma}_{ANCOVA}^2\right)$$

6 $${Y}_{j5}={Y}_{j1}+{\sigma}_{ANCOVA}\epsilon$$

Where *Y*_*j*5_ and *Y*_*j*1_ denote Week 26 and baseline value. *ϵ* is randomly sampled from standard normal distribution ***N***(0, 1). ${\sigma}_{ANCOVA}^2$ is the MSE from ANCOVA model based on completers adjusting for the same covariates, i.e. treatment and baseline for simulation studies.

##### J2R

For subjects in the study medication with missing Week 26 values, the visits before and after treatment discontinuation are modelled as a joint normal distribution with a mean of $\boldsymbol{E}\left({\boldsymbol{Y}}_j\right)=\left({\mu}_{j1},\dots, {\mu}_{jD-1,}\ {\mu}_{jD}^p,\dots, {\mu}_{j5}^p\right)$ and a covariance matrix such that

- the covariance up to last on-treatment visit, denoted as *D* − 1 is the same as the original covariance matrix of the study medication.
- The covariance of post-discontinuation visits conditional on observed data (i.e. visits prior to the treatment discontinuation) will be the same as the placebo group.

The imputation of the placebo group will follow MAR assumption and therefore no tweak on the joint distribution is needed for subjects with missing Week 26 values in the placebo group. However, the covariance matrix of subjects with missing Week 26 in the active group need to be derived using the constraints above.

The approach can be implemented by using the 5 macros [41] available on https://www.lshtm.ac.uk/.

##### Trimmed means

ANCOVA with adaptive trimming described in Permutt’s paper [38] is applied. Let *nm*_*p*_ and *nm*_*d*_ denote number of subjects with missing Week 26 in placebo and investigational drug respectively. After the data are ranked within each group, max(*nm*_*p*_, *nm*_*d*_) observations with missing Week 26 or lowest scores will be trimmed from each group. This type of trimming leads to minimal loss of information as well as removing all missing values. *p*-value and 95%CI are calculated using 10,000 permutations.

##### Mehrotra’s control-based method

Assuming the mean of subjects in the investigational drug group with missing Week 26 can be represented by the overall mean of placebo group, the treatment effect difference at Week 26 between investigational drug and placebo is written as

7 $$\delta ={p}_{d, com}\left({\hat{\mu}}_{d, com}-{\hat{\mu}}_p\right)$$

Where *p*_*d*, *com*_ is the proportion of subjects in the investigational drug group with non-missing Week 26, ${\hat{\mu}}_{d, com}$ is the MMRM mean at Week 26 among completers in the investigational drug group and ${\hat{\mu}}_p$ is the MMRM mean at Week 26 among placebo subjects. The variance and df are calculated using Kenward-Roger method.

#### Part 3. Power

We considered the same scenarios (Table 2) as the type-I error simulations except that the following effect size were utilized in dataset simulations to generate datasets under the alternative hypothesis:

1. $${\boldsymbol{\beta}}_{\boldsymbol{I}}=\left(0,-0.1,-0.2,-0.4,-0.5\right)^{\prime }$$
2. $${\boldsymbol{\beta}}_{\boldsymbol{I}}=\left(0,-0.1,-0.2,-0.25,-0.3\right)^{\prime }$$

**Fig. 4:**
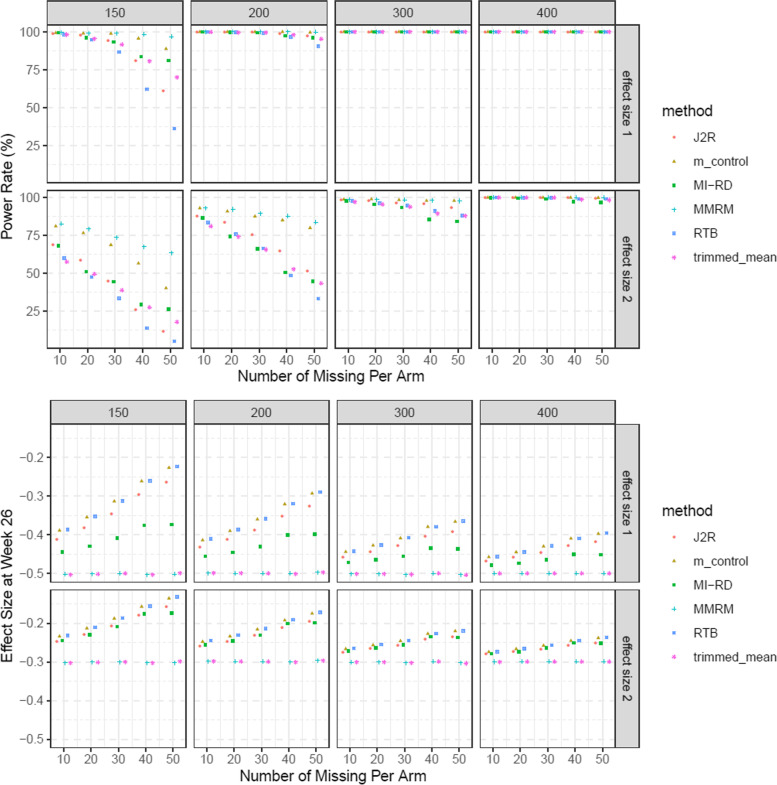
Power rate and Effect size (difference of treatment effect at Week 26) of all 6 methods with respect to different sample size: m_control denotes Mehrotra’s control-based method; effect size 1 and 2 denote the effect size 1) and 2)

In each scenario, 1000 datasets were simulated under the alternative hypothesis that the active group was superior to placebo (i.e. the last element of ***β***_***I***_ < 0 in both 1) and 2)) in reducing A1c at Week 26. In addition, RDs in the active treatment group were assumed to have an additional average worsening of 0.25 at Week 26 due to off-treatment period, compared to completers. i.e. ***Y***_***i***_ = ***β***_**0**_ + ***β***_***t***_ + ***β***_***I***_*trt*_*i*_ + ***β***_***MNAR***_*trt*_*i*_*I*{*i is RD*} + ***ε***_***i***_**.** The power rate was defined as the proportion of simulations with one-sided *p*-value significant at *α* = 0.025. The results were summarized in Results section and presented in Fig. 4.

#### Part 4. No enough RDs

If a study is not designed to collect the data of RDs, very likely it might end up with fewer RDs than the minimum cutoff identified in Part 1. We propose the following strategies for potential consideration. The type-I error rate and power rate were evaluated in contrast with RTB. Pros and cons will be further compared in the discussion section.

- *Approach 1*: The response variable in this case is transformed to log(y-a) (a > 0; pre-specified) first and the multiple imputation is implemented on the transformed scale. The imputed values are then transformed back to the original scale and hence they are ensured to be greater than a. However, this approach might result in extremely large values due to large MSE and exponential transformation. In this case, some post-processing steps [42] are needed, e.g. right truncation or its variations.
- *Approach 2:* Use the original MI-RD approach to impute missing values and then apply both left and right truncations or their variations to imputed values falling out of the range.

**Fig. 5:**
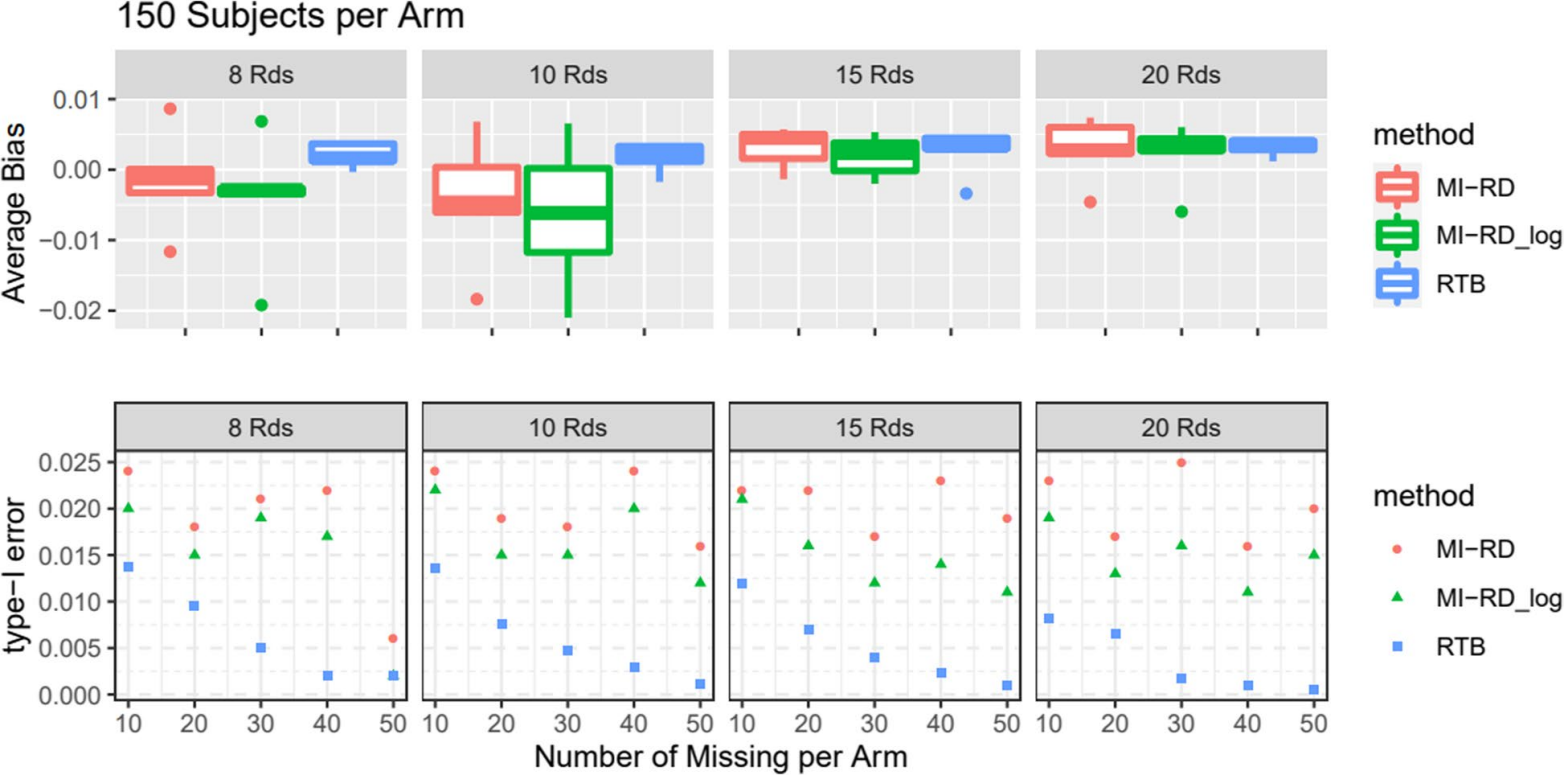
Type-I Error Rate and Average Bias with respect to Different Amount of Missingness and Different Number of RDs, for 150 Subjects per Arm

For the type-I error rate simulations, given an absolute amount of missingness per arm (10, 20, 30, 40, 50) and sample size per Arm (150, 200, 300, 400), different number of RDs per Arm (8, 10, 15, 20) were explored. The effect size ***β***_***I***_ = (0, −0.2, −0.4, −0.8, 0)′ was utilized. Results were presented in Fig. 5 and supplementary file.

**Fig. 6:**
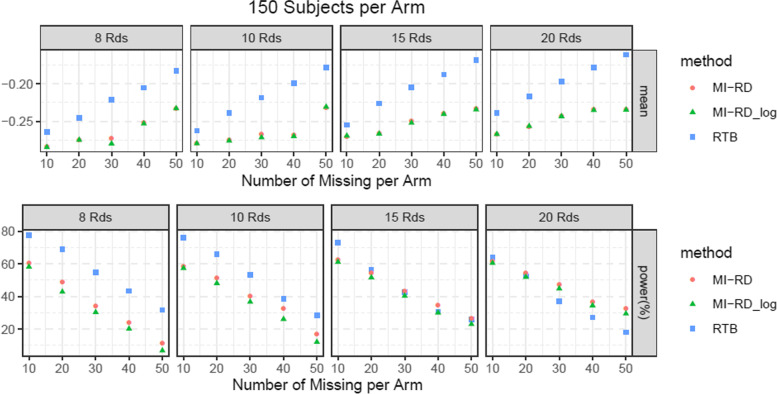
Power Rate and Effect Size Estimates with respect to Different Amount of Missingness and Different Number of RDs, for 150 Subjects per Arm

For the power rate simulations, we explored the same scenarios as type-I error simulations, except that effect size ***β***_***I***_ = (0, −0.1, −0.2, −0.25, −0.3)′ was utilized to simulate data under the alternative hypothesis. In addition, an average worsening of 0.15 for the Week 26 values of RDs in the active treatment group was applied. i.e. ***Y***_***i***_ = ***β***_**0**_ + ***β***_***t***_ + ***β***_***I***_*trt*_*i*_ + ***β***_***MNAR***_*trt*_*i*_*I*{*i is RD*} + ***ε***_***i***_ where ***β***_***MNAR***_ ***=*** (0, 0, 0, 0,  0.15)′**.** Results were presented in Fig. 6 and supplementary file.

### Post-hoc data analysis

Post-hoc analyses using this method, in contrast to MMRM, RTB, J2R, Mehrotra’s control-based and trimmed means were conducted on an unblinded dataset of a Pfizer phase III lipid-lowering study (NCT01968967). The primary endpoint of interest was change from baseline in low-density lipoprotein (LDL) at Week 52. There were 2099 patients in total (1051 in placebo; 1048 in the active treatment group), out of which 332 were RDs defined as subjects whose Week 52 values were collected at least 21 days after their last dose of treatment (166 in placebo; 166 in the active treatment group), 250 had missing values at Week 52 (131 in placebo; 119 in the active treatment group). Results of all three methods were summarized in Table 4.

**Table 4:** Application of the Following Methods to a Real Phase III Dataset

Method	Difference of treatment effect active vs placebo	95% CI	*p*-value
MI-RD	−40.58	[−43.92, −37.24]	<.0001
MMRM (off-treatment visits were set to missing, i.e. "hypothetical")	−50.33	[−53.23, −47.43]	<.0001
RTB (off-treatment visits were set to missing and hence imputed)	−38.97	[−41.90, −36.04]	<.0001
MMRM (including off-treatment visits, i.e. "TP")	−43.29	[−46.10, −40.49]	<.0001
J2R	−36.59	[−39.85, −33.1]	<.0001
Mehrotra’s control-based	−37.62	[−40.36, −34.88]	<.0001
Trimmed Means	−51.41	[−55.55, −47.27]	<.0001

## Results

### Simulation results

#### Best applicable scenarios

Given an amount of missingness per arm, the conclusion on the minimum number of RDs per arm doesn’t quite differ by the magnitude of effect size (***β***_***I***_: difference of treatment effect between test and reference treatment group) as reflected in Fig. 2. Generally, more missing data require more RDs, as more missing data require higher precision of the regression model built from RDs, so as to ensure all imputed values would fall in the appropriate range. Given the number of missing data per arm, the biggest minimum number of RDs across all three effect size scenarios was summarized and used as input for type-I error and power rate simulations (column 2 of Table 1).

#### Type-I error

Figure 3 shows the type-I error rate of MI-RD is well controlled across all scenarios. The MMRM has well-controlled type-I error rate for most scenarios. Mehrotra’s control-based has slightly deflated type-I error rate for some scenarios. e.g., a study with more than 30% missing data should be cautious about using this control-based method as it might lead to deflated type-I error rate. The rest of the methods (J2R, RTB, adaptive trimmed means) all have deflated type-I error rate with similar pattern: 1) given a sample size and an effect size, the type-I error becomes more deflated with more missing data; 2) It’s also evident that given an amount of missingness (e.g. 20 missing per arm), the deflation of type-I error has less impact on a bigger sample size (e.g. 400 per arm vs 150 per arm). Out of the three methods (J2R, RTB, adaptive trimmed means), adaptive trimmed means seems to be the most conservative, followed by RTB and J2R. All 6 methods are considered unbiased in terms of the fact that data are simulated under the null hypothesis that there’s no difference of treatment effect between active and placebo at Week 26.

#### Power

Generally, the MMRM has the highest power rate, followed by Mehrotra’s control-based method and MI-RD. RTB and J2R are the most conservative. The difference gradually diminishes with bigger sample size. In terms of effect size estimate, MMRM and trimmed means return the biggest effect size estimate due to the fact that retrieved dropouts’ off-treatment visits are not used in the analysis. Estimates of J2R, Mehrotra’s control-based and RTB are the smallest due to conservative assumptions of either returning to baseline or returning to distribution of control group. MI-RD falls in between (see Fig. 4). The benchmark for effect size (difference between active and placebo) at Week 26 is ***β***_***I***_[5] + ***β***_***MNAR***_[5]∗ proportion of RD, assuming missing data follows MAR pattern. But since our proposed method is based on one MNAR assumption leading to effect attenuation like other MNAR approaches (J2R, Mehrotra’s control-based, RTB), the estimated effect size at Week 26 is smaller than this benchmark, as reflected in Fig. 4. It’s noteworthy that our proposed method is the least conservative among all MNAR approaches explored. Because RDs in the active treatment group are assumed to have some level of worsening compared to completers in the same group after treatment discontinuation and their off-treatment primary visits are included in the analysis of MI-RD, an estimated effect size smaller than the simulated effect size is expected.

#### No enough RDs

##### Type-I error rate

Approach 2 with no log transformation best preserves the type-I error rate among the three methods, while RTB is the most conservative in most scenarios (Fig. 5 and supplementary file). With bigger sample size, the difference in type-I error rate across all 3 methods become smaller (e.g. 300, 400 vs 150 per arm). All three methods are considered unbiased.

##### Power rate

Approach 1 and 2 yield pretty much the same power rate and effect size estimates for each scenario. They approximately have higher power than RTB in scenarios with 150–200 subjects per arm and 15–20 RDs per arm. RTB can be slightly more powerful in scenarios with 150–200 subjects per arm and 8–10 RDs or 300 subjects per arm (Fig. 6 and supplementary file). Consistent with previous findings, effect size estimates of RTB is smaller due to more conservative assumption. It is also self-explanatory that more missing data further attenuates the effect size.

### Post-hoc data analysis results

Consistent with simulation results: MMRM under hypothetical estimand and trimmed means return the largest effect size estimate, followed by MMRM (TP estimand), MI-RD, RTB, Mehrotra’s control-based and J2R. RTB, Mehrotra’s control-based and J2R yield the most conservative estimate of effect size. All methods return *p* values of less than 0.0001.

## Discussion

There have been considerable debates over the past few decades on which assumptions are generally acceptable for MI analyses or analyses that don’t need explicit imputation of missing data such as MMRM. MAR assumptions do hold in certain scenarios while MNAR works better in other cases. For instance, a subject in the active group who discontinued the trial due to adverse events may no longer keep the treatment effect after discontinuation, whereas it might be fine to assume MAR for another subject who discontinued the trial due to moving to another city. An overall assumption of MNAR is becoming the future trend because it can determine how robust primary conclusion is, by deviating from favorable assumptions. A very noteworthy feature of our proposed approach is it balances the controversy between the two totally opposite perspectives.

For our proposed method, we simulated different amount of missing data under different sample size, to account for different missing/discontinuation rate (ranging from 2 to 33%) with respect to trials in different therapeutic areas /clinical stages. The rationale of simulating absolute missing data is that the minimum number of RDs is directly related to the absolute amount of missing data. Results of Part1 provide insights to sponsors interested in this approach so that a study can be designed to collect sufficient retrieved dropouts. It clearly has demonstrated minimum number of RDs are directly related to number of missing values. MI is not a new topic, but sometimes statisticians tend to skip checking the validity of imputed values (e.g. if an imputed value is in the right range), which can result in misleading and biased estimates when such values are included in the analysis. Some people may argue imposing the criteria of having all imputed values within plausible range is too strict and unnecessary. On one hand, extreme values certainly will distort the results if they are way too extreme (e.g. an imputed value of > 1000 for A1c(%)). But on the other hand, I agree moderately increasing the tolerance with justification on unbiased estimates, no loss of efficiency, etc. can be considered. For pivotal trials, it’s good practice to obtain the nod from regulatory agency on the most appropriate strategy during protocol/SAP review.

Although traditional MAR-based MI usually includes all scheduled visits ranging from baseline to the primary visit in the imputation [43], we propose only baseline, last on-treatment visit are included as regressors of the imputation, based on regulatory feedback. The rationale is with only a small subset (RDs) used as the imputation basis, it’s not very possible to keep all or majority of imputed values within plausible range if all intermediate visits are included. Our proposed imputation approach also simplifies the regression-based imputation due to its inherent monotone structure [44]. We suggest a total of 100 imputations for the following considerations: more imputations can effectively prevent power falloff [24], but more than 100 imputations are usually not computationally affordable for large datasets. For some methods involving very intensive computation, regulatory agencies might even agree with < 50 imputations, based on our past regulatory interactions.

Classifying a subject as a RD or not directly relies on the definition of intercurrent events. Generally speaking, every clinical study defines intercurrent events somewhat differently. For instance, recent glycemic clinical studies primarily define treatment discontinuation or initiation of rescue therapy as intercurrent events [32–35, 45]. Some lipid-lowering clinical programs might only define treatment discontinuation as intercurrent events [46], like the data analysis application. Therefore, in the former case, RDs are defined as subjects with primary endpoint collected off treatment or collected after initiation of rescue therapy. While in the latter, RDs are defined as subjects with primary endpoint collected off treatment.

With enough RDs, the MI-RD approach has quite a few advantages: 1) it includes more data in the analysis under the ITT principle, as opposed to approaches in the paradigm of hypothetical estimand which excludes observations that occur post occurrence of intercurrent events. 2) It well preserves the type-I error rate, compared to other commonly used MNAR methods. 3) the attenuation effect of RDs are accounted for in the effect size estimation, in contrast to hypothetical estimand approaches which exclude RDs’ off-treatment visits from the analysis.

As for the two modified approaches for scenarios with insufficient RDs, approach 2 has better performance than approach 1. They both have pretty much the same simulation results but approach 2 preserves the original distribution of the endpoint in the imputation of missing data. Furthermore, due to the log transformation in approach 1, more extremely large values from imputation are generated and truncated. However, since the modified MI-RD approaches only work better in certain scenarios and there’s always concern that post-processing steps might lead to biased estimates [31], sponsors should be more open-minded to other approaches especially when the trial is not designed to collect retrieved dropouts.

Similar to power rate calculation using simulations, sample size can be estimated starting from an initial sample size along with the scheme of line/grid search. This will inform the sponsor as how many subjects need to be enrolled to achieve the pre-specified power, when MI-RD is planned as the primary analysis.

The less granular MI (i.e. implemented within groups defined by treatment group) is recommended, unless a trial has an enormous amount of RDs. Of all above methods that we have compared to, J2R is the most computationally intensive. MI-RD, however, can be implemented very efficiently in commonly used statistical analysis programs.

Using similar strategy, this approach can be extended to other types of endpoints, such as binary, multinomial or count endpoints. Like in generalized linear model theory, link function *g*(.) will be applied to eq. (1), (2), (4) which will cover imputation and estimation phases, in the form of *g*(*E*(*Y*)). It’s noteworthy if continuous endpoints are collected and available despite a non-continuous endpoint is of interest in the analysis, it’s recommended to use continuous endpoint for imputation phase (1)/ (4). For instance, an endpoint of interest is A1c < 7% with A1c data available. Then continuous A1c should be used for imputation phase although binary A1c < 7% will be used in estimation and hypothesis testing phases. This approach can also be extended to time to event endpoints with or without parametric assumptions. We applied it to time to event endpoints of an outcome trial in a recent FDA submission. One advantage of such application is there will be very little concern on collecting sufficient retrieved dropouts due to relatively large sample size of an outcome trial. The statistical methodology details are being developed in a separate manuscript.

As shown in our simulation studies: results based on different assumptions don’t vary much when there are very few missing data. Any efforts of minimizing missing data for clinical trials should be encouraged such as developing strategies or surveys to collect post-discontinuation information, because currently subjects’ behavior after their discontinuation from the trials is largely unknown. Post-discontinuation information is critical in terms of predicting which assumption is the most appropriate for a particular clinical trial, and hopefully such improvements will be realized through scientific collaboration in the future.

## Conclusion

This proposed MI approach is best applicable to trials designed to collect retrieved dropouts. Because it fully aligns with the ITT principle and is based on very reasonable MNAR assumption that the treatment effect of missing data follows the same distribution of retrieved dropouts in the same treatment group after adjustment for baseline and last on-treatment values, this approach can be used as primary analysis. The implementation is very straightforward and computer efficient, such as in SAS and R. Similar to power rate calculation, sample size can be estimated using simulation studies for studies interested in pre-specifying this approach as primary analysis. This approach can also be extended to survival and binary endpoints using parametric or semi-parametric models. When a trial doesn’t have enough RD, other approaches should be given priority to in terms of primary analysis especially when the power of transformed MI-RD is not satisfactory (reference the power plots in the supplementary files).

## Supplementary Information

**Additional file 1.**

## Data Availability

The dataset used in the data analysis won’t be available from corresponding author, due to Pfizer’s policy and obligation to protect patients’ privacy. The datasets used in the simulation studies can be made available from corresponding author on reasonable request.

## Abbreviations

MAR: Missing at random
MNAR: Missing not at random
T2D: Type 2 diabetes
MMRM: Mixed model repeated measurements
cLDA: Constrained longitudinal data analysis
MI: Multiple imputation
J2R: Jump to reference
BOCF: Baseline observation carried forward
RTB: Return to baseline
RD: Retrieved dropout
ANCOVA: Analysis of covariance
ITT: Intent to treat
MSE: Mean squared error

## References

[1] Larsen ML, Hørder M, Mogensen EF (1990). Effect of long-term monitoring of glycosylated hemoglobin levels in insulin-dependent diabetes mellitus. N Engl J Med.

[2] Caveney EJ, Cohen OJ (2011). Diabetes and biomarkers. J Diabetes Sci Technol.

[3] U.S. Department of Health and Human Services, Food and Drug Administration, Center for Drug Evaluation and Research. Guidance for industry. diabetes mellitus: Developing drugs and therapeutic biologics for treatment and prevention. 2010.

[4] Dziura JD, Post LA, Zhao Q, Fu Z, Peduzzi P (2013). Strategies for dealing with missing data in clinical trials: from design to analysis. Yale J Biol Med.

[5] Liu GF, Lu K, Mogg R, Mallick M, Mehrotra DV (2009). Should baseline be a covariate or dependent variable in analyses of change from baseline in clinical trials?. Statist Med..

[6] EMA/CPMP/EWP/1776/99 Rev. 1. Guideline on missing data in confirmatory clinical trials. 2010.

[7] FDA (2021). E9(R1) statistical principles for clinical trials: Addendum: Estimands and sensitivity analysis in clinical trials.

[8] Carpenter J, Kenward M. Multiple imputation and its application: Wiley; 2012. 10.1002/9781119942283.

[9] Carpenter JR, Kenward MG (2007). Missing data in randomised controlled trials: a practical guide.

[10] Liu-Seifert H, Zhang S, D'Souza D, Skljarevski V (2010). A closer look at the baseline-observation-carried-forward (BOCF). Patient Preference Adherence.

[11] Roderick JAL (1993). Pattern-mixture models for multivariate incomplete data. J Am Stat Assoc.

[12] Roderick JAL (1994). A class of pattern-mixture models for normal incomplete data. Biometrika..

[13] Ratitch B, O'Kelly M, Tosiello R (2013). Missing data in clinical trials: from clinical assumptions to statistical analysis using pattern mixture models. Pharmaceut Statist.

[14] Carpenter JR, Roger JH, Kenward MG (2013). Analysis of longitudinal trials with protocol deviation: a framework for relevant, accessible assumptions, and inference via multiple imputation. J Biopharm Stat.

[15] Zhang Y, Golm G, Liu G (2020). A likelihood-based approach for the analysis of longitudinal clinical trials with return-to-baseline imputation. Stat Biosci.

[16] Shao J, Jordan DC, Pritchett YL (2009). Baseline observation carry forward: reasoning, properties, and practical issues. J Biopharm Stat.

[17] Siddiqui O, Ali MW (1998). A comparison of the random-effects pattern mixture model with last-observation-carried-forward(locf) analysis in longitudinal clinical trials with dropouts. J Biopharm Stat.

[18] Lyons TJ, Basu A (2012). Biomarkers in diabetes: hemoglobin A1c, vascular and tissue markers. Transl Res.

[19] Yan X, Lee S, Li N (2009). Missing data handling methods in medical device clinical trials. J Biopharm Stat.

[20] Lipkovich I, Ratitch B, O'Kelly M (2016). Sensitivity to censored-at-random assumption in the analysis of time-to-event endpoints. Pharmaceut Statist..

[21] Farlow M, Potkin S, Koumaras B, Veach J, Mirski D (2003). Analysis of outcome in retrieved dropout patients in a rivastigmine vs placebo, 26-week, alzheimer disease trial. Arch Neurol.

[22] Chen Q, Chen M, Ohlssen D, Ibrahim JG (2013). Bayesian modeling and inference for clinical trials with partial retrieved data following dropout. Statist Med.

[23] Pampaka M, Hutcheson G, Williams J (2016). Handling missing data: analysis of a challenging data set using multiple imputation. null..

[24] Graham JW, Olchowski AE, Gilreath TD (2007). How many imputations are really needed? Some practical clarifications of multiple imputation theory. Prev Sci.

[25] Akacha M, Bretz F, Ohlssen D, Rosenkranz G, Schmidli H (2017). Estimands and their role in clinical trials. Stat Biopharm Res.

[26] Little RJ, D'Agostino R, Cohen ML (2012). The prevention and treatment of missing data in clinical trials. N Engl J Med.

[27] STEGLATRO™ (ertugliflozin) Tablets, for oral use initial U.S. approval: 2017. Updated 2017.

[28] STEGLUJAN™ (ertugliflozin and sitagliptin) tablets, for oral use initial U.S. approval: 2017. Updated 2017.

[29] Sakyi A, Laing E, Ephraim R, Asibey O, Sadique O (2015). Evaluation of analytical errors in a clinical chemistry laboratory: a 3 year experience. Ann Med Health Sci Res.

[30] Rubin D (1987). Multiple imputation for nonresponse in surveys.

[31] Rodwell L, Lee KJ, Romaniuk H, Carlin JB (2014). Comparison of methods for imputing limited-range variables: a simulation study. BMC Med Res Methodol.

[32] Lingvay I, Desouza CV, Lalic KS (2018). A 26-week randomized controlled trial of semaglutide once daily versus liraglutide and placebo in patients with type 2 diabetes suboptimally controlled on diet and exercise with or without metformin. Diabetes Care.

[33] Rosenstock J, Frias J, Páll D (2018). Effect of ertugliflozin on glucose control, body weight, blood pressure and bone density in type 2 diabetes mellitus inadequately controlled on metformin monotherapy (VERTIS MET). Diabetes Obes Metab.

[34] Russell-Jones D, Cuddihy RM, Hanefeld M (2012). Efficacy and safety of exenatide once weekly versus metformin, pioglitazone, and sitagliptin used as monotherapy in drug-naive patients with type 2 diabetes (DURATION-4). Diabetes Care.

[35] Terra SG, Focht K, Davies M (2017). Phase III, efficacy and safety study of ertugliflozin monotherapy in people with type 2 diabetes mellitus inadequately controlled with diet and exercise alone. Diabetes Obes Metab.

[36] Low hemoglobin a1c in nondiabetic adults. 10.2337/dc11-2531.

[37] Carson AP, Fox CS, McGuire DK (2010). Low hemoglobin A1c and risk of all-cause mortality among US adults without diabetes. Circ Cardiovasc Qual Outcomes.

[38] Permutt T, Li F (2017). Trimmed means for symptom trials with dropouts. Pharm Stat.

[39] Mehrotra DV, Liu F, Permutt T (2017). Missing data in clinical trials: control-based mean imputation and sensitivity analysis. Pharm Stat.

[40] Cannon CP, McGuire DK, Pratley R (2018). Design and baseline characteristics of the eValuation of ERTugliflozin effIcacy and safety CardioVascular outcomes trial (VERTIS-CV). Am Heart J.

[41] Cro S, Morris TP, Kenward MG, Carpenter JR (2020). Sensitivity analysis for clinical trials with missing continuous outcome data using controlled multiple imputation: a practical guide. Stat Med.

[42] Yulei H (2010). Missing data analysis using multiple imputation. Circ Cardiovasc Qual Outcomes.

[43] Pedersen AB, Mikkelsen EM, Cronin-Fenton D (2017). Missing data and multiple imputation in clinical epidemiological research. Clinical epidemiology.

[44] Horton NJ, Kleinman KP (2007). Much ado about nothing: A comparison of missing data methods and software to fit incomplete data regression models. Am Stat.

[45] Strojek K, Pandey AS, Dell V, et al. Efficacy and safety of ertugliflozin in patients with diabetes mellitus inadequately controlled by sulfonylurea monotherapy: a substudy of VERTIS CV. Diabetes Ther. 2021; 10.1007/s13300-021-01018-w. 10.1007/s13300-021-01018-w PMC7994479 33694093

[46] Ridker PM, Amarenco P, Brunell R (2016). Evaluating bococizumab, a monoclonal antibody to PCSK9, on lipid levels and clinical events in broad patient groups with and without prior cardiovascular events: rationale and design of the studies of PCSK9 inhibition and the reduction of vascular events (SPIRE) lipid lowering and SPIRE cardiovascular outcomes trials. Am Heart J.

