# Supplementary material for: Impute the missing data using retrieved dropouts
Source: BMC Med Res Methodol. 2022 Mar 27;22:82. doi: 10.1186/s12874-022-01509-9 (PMC8962050; doi:10.1186/s12874-022-01509-9)
Supplement: Supplementary file 1 — Additional file 1. [file 12874_2022_1509_MOESM1_ESM.docx]

# Code

- **MI-RD SAS Macro and demo**

*Author: Shuai Wang (shuai1107@hotmail.com)

***************************************;

*Using simplified imputation phase: by treatment group;

*when there are no enough retrieved dropouts, the macro will perform return to baseline analysis;

%macro MI_rd(indata=,

avisitn=/*Numeric visit variable*/,

base=,

treatment=,

visitfl=/*visit flag, e.g. ablfl='Y' or avisitn in (6,12,18,26)*/,

last_ontrt_var=/*last on treatment value*/,

classvar=/*categorical variables used in the class statement of ANCOVA estimation step*/,

modelterms=/*covariates used in ANCOVA*/,

seed=/*MI seed*/,

nimpute=,

rd=/*retrieve dropout status indicator variable: 1=retrieved dropout; 0=non-retrieved dropout*/,

seed_rtb=/*seed for RTB imputation*/,

add_var=/*variables used in the by statement of proc transpose in addition to subjid, trt01pn, trt01p, base and rd*/,

primary_time=/*v+timepoint, e.g. v18 or v26*/

);

data adeff;

set &indata;

if &visitfl;

if &base ne .;

run;

proc sort data=adeff nodupkey;

by subjid &avisitn;

run;

proc sort data=adeff;

by subjid &treatment &base &add_var &rd;

run;

proc transpose data=adeff prefix=v out=adeff_t;

by subjid &treatment &base &add_var &rd/*retrieve dropout status*/;

id &avisitn;

var aval;

run;

*obtain the sd from ancova;

proc mixed data=adeff_t (where=(&rd ne 1));

class &classvar;

model &primary_time=&modelterms/ddfm=kr s;

ods output CovParms=covParms;/*this is the variance*/

run;

data _null_;

set covparms;

sd=sqrt(Estimate);/*square root is needed*/

call symput ('sd', sd);

run;

%put &sd;

data adeff_t2;

set adeff_t;

if &rd=1 or &primary_time=.;

run;

data adeff_rd;

set adeff_t2;

if &rd = 1;

run;

proc sort data=adeff_t2;

by &treatment;

run;

proc freq data=adeff_rd;

tables &treatment/out=adeff_rd_pt1_freq1 sparse;

run;

data adeff_rd_pt1_freq1;

set adeff_rd_pt1_freq1;

drop percent;

rename count=count1;

run;

proc freq data=adeff_t2 (where=(&rd=0));

tables &treatment/out=adeff_rd_pt1_freq0 sparse;

run;

data adeff_rd_pt1_freq0;

set adeff_rd_pt1_freq0;

drop percent;

rename count=count0;

run;

data adeff_rd_pt1_freq;

merge adeff_rd_pt1_freq0 adeff_rd_pt1_freq1;

by &treatment;

run;

data adeff_rd_pt1_v2;

merge adeff_t2 adeff_rd_pt1_freq;

by &treatment ;

run;

proc sql;

select count(distinct subjid) into: n_mird from adeff_rd_pt1_v2

where count1>4 and count0>0 and &primary_time =. ;

proc mi data=adeff_rd_pt1_v2(where=(count1>4 and count0>0)) out=adeff_mi nimpute=&nimpute seed=&seed;

by &treatment ;

monotone reg;

var &base &last_ontrt_var &primary_time;

run;

proc sort data=adeff_mi out=adeff_mi_sort nodupkey;

by &treatment _imputation_;

run;

data adeff_mi_sort;

set adeff_mi_sort;

by &treatment;

if last.&treatment then output;

run;

proc sql;

select min(_imputation_) into: min_imp from adeff_mi_sort;

quit;

%let footnote_missing="Imputed using MI-RD.";

%if %sysevalf(&min_imp<&nimpute) %then %do;

%let footnote_missing="Imputed using RTB.";

/*In RTB implementation, for subjects missing at the primary_time, impute using RTB;

For completers, just repeat their value at the primary time for a total of nimpute times */

data adeff_mi_rtb;

set adeff_t;

do i=1 to &nimpute;

_imputation_=i;

sd=&sd;

seed=&seed_rtb;

r=rannor(seed);

if &primary_time=. then vnew=&base+sd*r;

else vnew=&primary_time;

output;

end;

drop i;

run;

data adeff_all_final;

set adeff_mi_rtb;

*drop &primary_time;

&primary_time=vnew;

run;

%end;

%else %do;

proc sql;

create table adeff_comp as select * from adeff_t

where adeff_t.subjid not in (select subjid from adeff_mi);

quit;

data adeff_comp_mi;

set adeff_comp;

do i=1 to &nimpute;

_imputation_=i;

output;

end;

run;

data adeff_all_final;

set adeff_comp_mi adeff_mi;

run;

%end;

proc sort data=adeff_all_final;

by _imputation_;

run;

data adeff_all_final;

set adeff_all_final;

chg=&primary_time-&base;

run;

/*Analyze the assembled dataset using ANCOVA by _imputation_*/

proc mixed data=adeff_all_final;

class &classvar;

by _imputation_;

model chg=&modelterms;

lsmeans &treatment;

estimate "active vs placebo" &treatment -1 1;

ods output estimates=est lsmeans=lsmeans ;

run;

proc sort data=est;

by label;

run;

/*Using Rubin's rule to combine the results*/

proc mianalyze data=est;

by label;

modeleffects estimate;

stderr stderr;

ods output parameterestimates=est_mi;

run;

proc sort data=lsmeans;

by &treatment;

run;

proc mianalyze data=lsmeans;

by &treatment;

modeleffects estimate;

stderr stderr;

ods output parameterestimates=lsmeans_mi;

run;

%put &footnote_missing;

%mend;

*demo is imported using simulated dataset A_long;

%include "mird_customized.sas";

options mprint mlogic;

%MI_rd(indata=demo,

avisitn=avisitn/*Numeric visit variable*/,

base=base,

treatment=trt01pn,

visitfl=avisitn in (6, 12, 18, 26)/*visit flag, e.g. ablfl='Y' or avisitn in (6,12,18,26)*/,

last_ontrt_var=last_ontrtval,

classvar=trt01pn/*categorical variables used in the class statement of ANCOVA estimation step*/,

modelterms=base trt01pn/*covariates used in ANCOVA*/,

seed=321/*MI seed*/,

nimpute=5,

rd=rd/*retrieve dropout status indicator variable: 1=retrieved dropout; 0=non-retrieved dropout*/,

seed_rtb=135/*seed for RTB imputation*/,

add_var=last_ontrtval/*variables used in the by statement of proc transpose in addition to subjid, trt01pn, trt01p, base and rd*/,

primary_time=v26/*v+timepoint, e.g. v18 or v26*/

);

- **Example of dataset simulation**

library(MASS)

N=1000

subjid=100000+(1:N)

trt01pn=rep(c(0,1), each=(N/2))

trt01p=rep(c("placebo","active"), each=(N/2))

#simulate rd and missing

#5% rd

#5% missing

set.seed(999)

rd_miss_idx=sample(1:N, (N*0.1))

rd_idx=sample(rd_miss_idx, (N*0.05))

miss_idx=rd_miss_idx[!(rd_miss_idx %in% rd_idx)]

#construct a data frame with subjid, trt01pn, trt01p, rd(=0/1), miss(=0/1), base, aval, avisitn, last on-treatment visit

A=data.frame(subjid, trt01pn, trt01p, rd=rep(0, N), miss=rep(0, N), base=rep(NA, N), aval=rep(NA, N))

A[rd_idx, "rd"]=1

A[miss_idx, "miss"]=1

beta_i=c(0, -0.05, -0.1, -0.2, -0.25)

beta_t=c(0, -0.01, -0.05, -0.1, -0.2)

beta_mnar=c(0, 0, 0, 0, 0.25)

varmat=matrix(0.6, 5, 5)

diag(varmat)=1

A_long=NULL

for (i in 1:N){

y_main=8.5+beta_t+beta_i*as.numeric(i>(N/2))+beta_mnar*as.numeric(i>(N/2))*as.numeric(A[i, "rd"]==1)

y_i=mvrnorm(1, y_main, varmat)

subj_full=do.call(rbind, replicate(5, A[i,], simplify=FALSE))

subj_full$base=y_i[1]

subj_full$aval=y_i

subj_full$avisitn=c(0, 6, 12, 18, 26)

subj_full$last_ontrt=26

subj_full$last_ontrtval=y_i[5]

if (A[i, "miss"]==1){

#simulate last on-treatment visit to be either row 2, 3, 4

miss_r=sample(c(2,3,4),1)

#only visits till last on-treatment visit are kept to mimic clinical trial data

subj_full=subj_full[1:miss_r, ]

subj_full$last_ontrt=subj_full$avisitn[miss_r]

subj_full$last_ontrtval=y_i[miss_r]

}

if (A[i, "rd"]==1){

#simulate last on-treatment visit to be either row 2, 3, 4

miss_r=sample(c(2,3,4),1)

subj_full$last_ontrt=subj_full$avisitn[miss_r]

subj_full$last_ontrtval=y_i[miss_r]

}

A_long=rbind(A_long, subj_full)

}

# Supplementary Figures


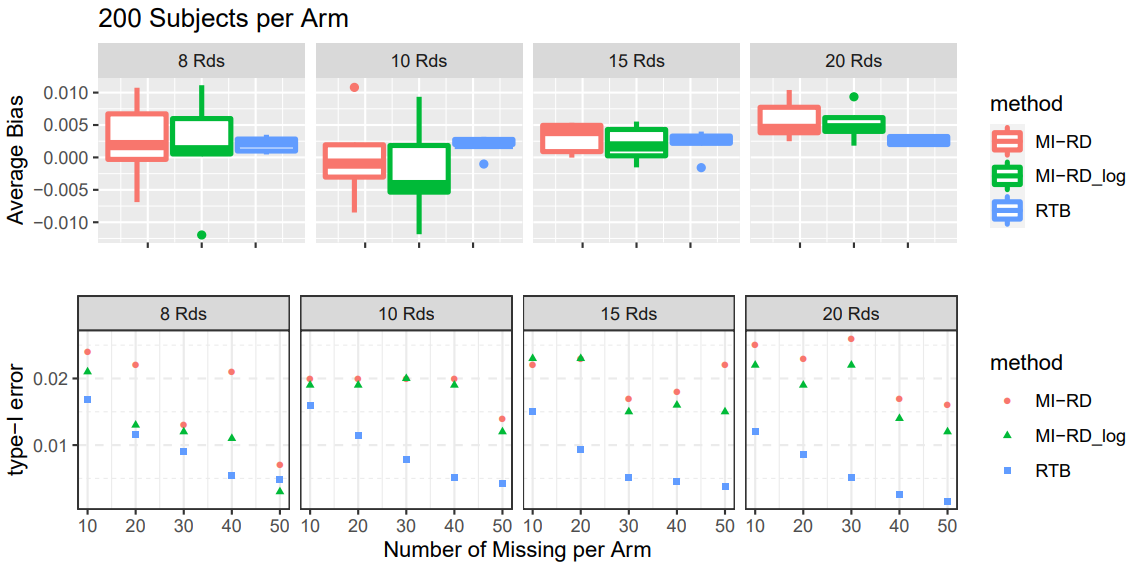


**Supplementary Figure 1.** **Type-I Error Rate and Average Bias with respect to Different Amount of Missingness and Different Number of RDs, for 200 Subjects per Arm**


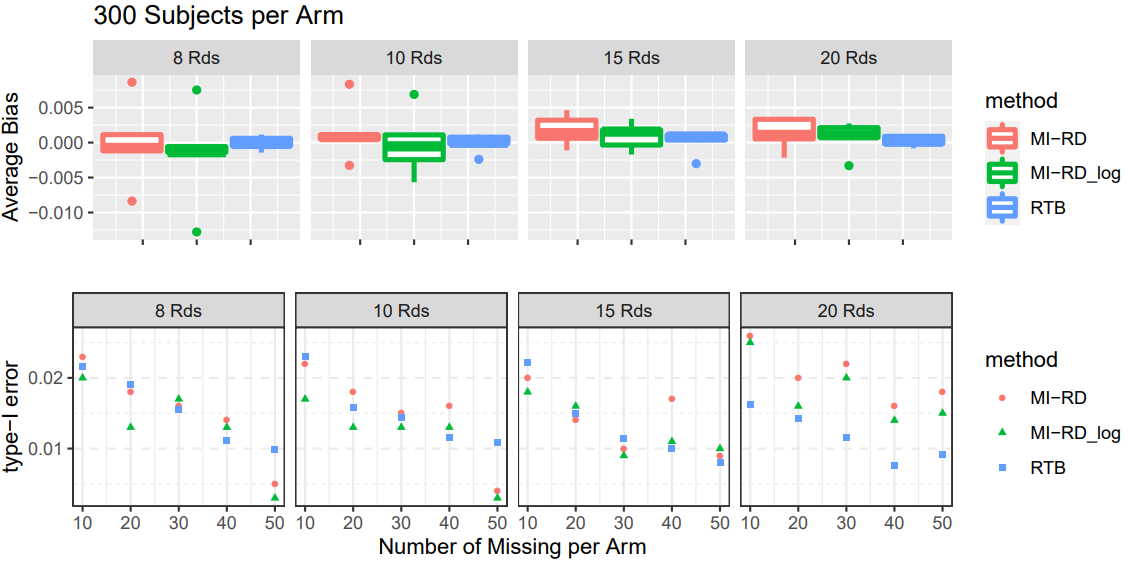


**Supplementary Figure 2.** **Type-I Error Rate and Average Bias with respect to Different Amount of Missingness and Different Number of RDs, for 300 Subjects per Arm**


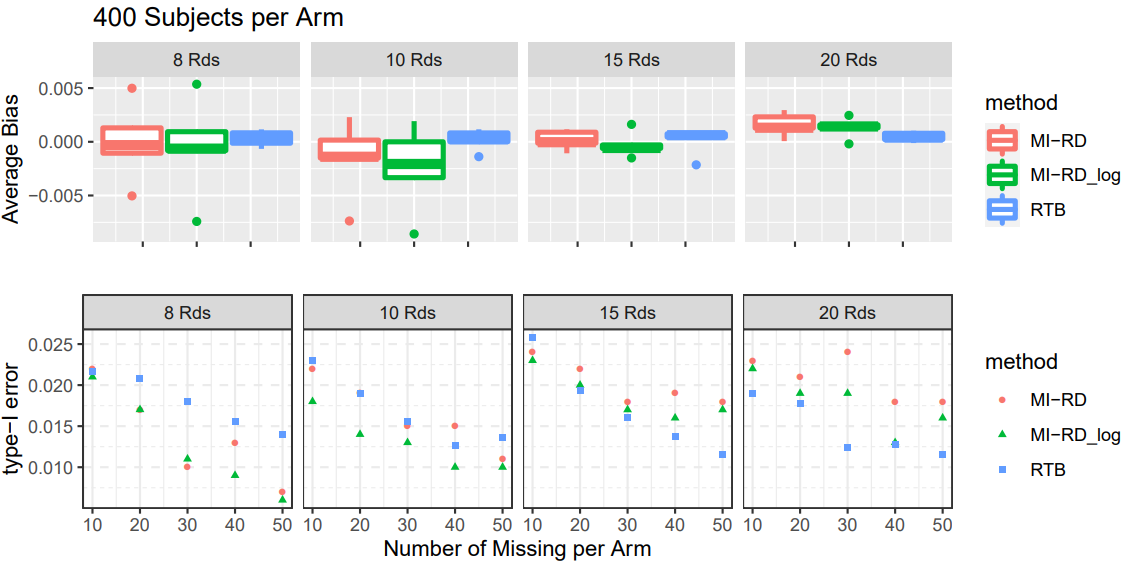


**Supplementary Figure** **3.** **Type-I Error Rate and Average Bias with respect to Different Amount of Missingness and Different Number of RDs, for 400 Subjects per Arm**


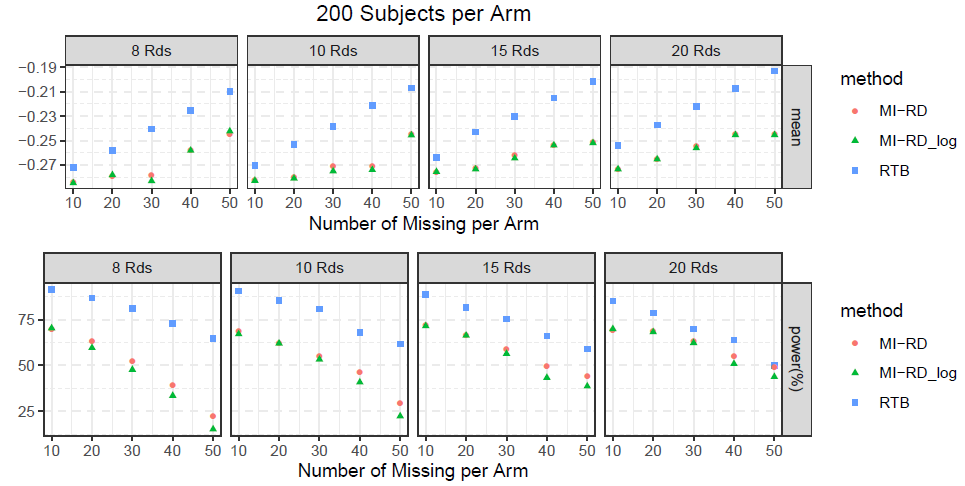


**Supplementary Figure 4.** **Power Rate and Effect Size Estimates with respect to Different Amount of Missingness and Different Number of RDs, for 200 Subjects per Arm**


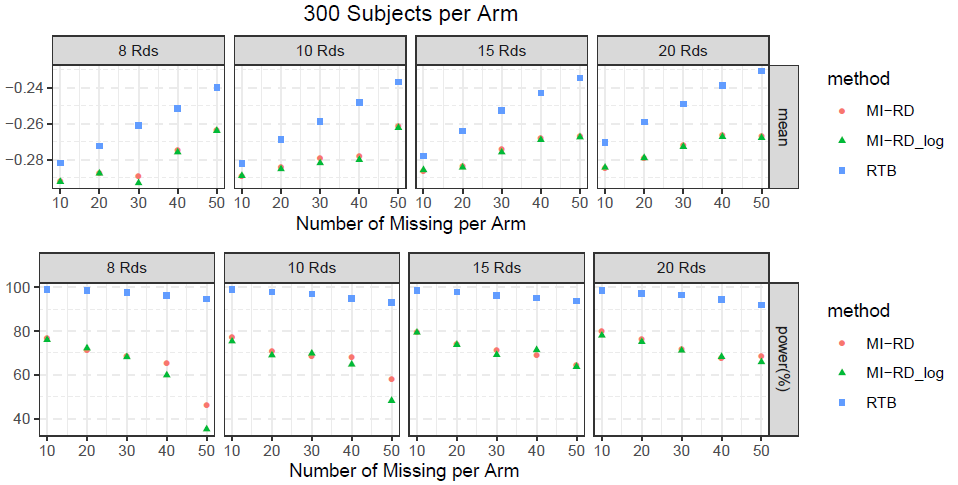


**Supplementary Figure 5.** **Power Rate and Effect Size Estimates with respect to Different Amount of Missingness and Different Number of RDs, for 300 Subjects per Arm**


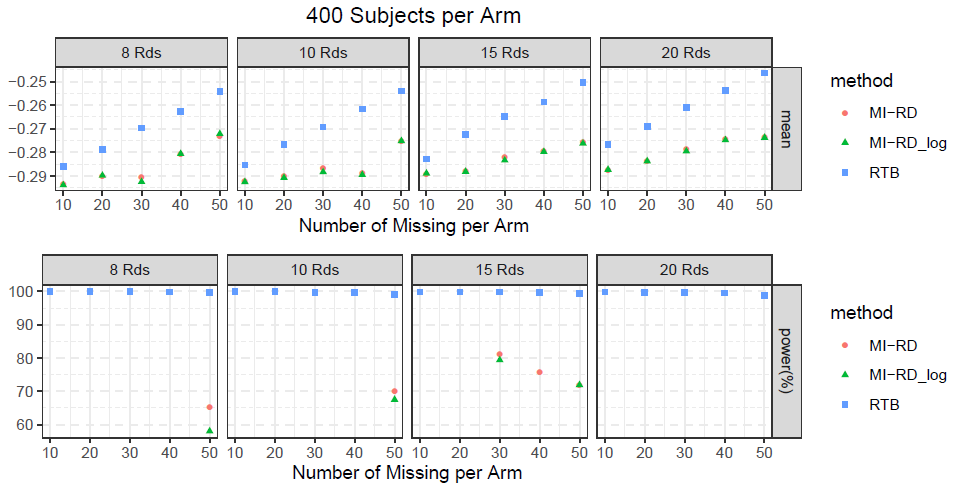


**Supplementary Figure 6.** **Power Rate and Effect Size Estimates with respect to Different Amount of Missingness and Different Number of RDs, for 400 Subjects per Arm**
